# Prospective mental imagery in patients with major depressive disorder or anxiety disorders

**DOI:** 10.1016/j.janxdis.2011.06.012

**Published:** 2011-12

**Authors:** Nexhmedin Morina, Catherine Deeprose, Christina Pusowski, Marina Schmid, Emily A. Holmes

**Affiliations:** a Department of Clinical Psychology, University of Amsterdam, The Netherlands; b Department of Psychiatry, University of Oxford, UK; c Department of Clinical Psychology, University of Frankfurt, Germany

**Keywords:** Depression, Anxiety, Mental imagery, Prospective cognition, Intrusions, Flashforwards

## Abstract

Prospective negative cognitions are suggested to play an important role in maintaining anxiety disorders and major depressive disorder (MDD). However, little is known about positive prospective mental imagery. This study investigated differences in prospective mental imagery among 27 patients with anxiety disorders, 24 patients with MDD, and 32 control participants. Measures of both deliberately generated and intrusive imagery were completed. Results indicated that both patients with anxiety disorders and those with MDD provided poorer vividness ratings for deliberately generated prospective positive scenarios compared to the control group. Patients with anxiety disorders showed a greater ability to vividly generate imagery for prospective negative scenarios than both patients with MDD and control participants. Finally, both clinical groups reported greater levels of intrusive prospective imagery of personally-relevant events as compared to the control group. The current findings underline the necessity to target prospective positive mental imagery in treating MDD and anxiety disorders.

## Highlights

► We examined differences in prospective mental imagery (PMI) in anxiety and depression. ► Patients wish depression or anxiety disorders reported less vivid positive PMI than controls. ► Patients with anxiety disorders reported more vivid negative PMI than controls. ► Both clinical groups reported greater levels of intrusive PMI than controls.

## 1 Introduction

An extensive body of research has demonstrated support for cognitive theories indicating that cognitions can play a significant role in the development and maintenance of major depressive disorder (MDD) and anxiety disorders (Craske et al., 2009; Gotlib & Joormann, 2010). Cognitions are conceptualized as taking the form of verbal thoughts or mental images (Beck, 1976) although the focus has traditionally been on verbal thoughts. In both depression and anxiety, faulty cognitive processes include difficulties in shifting attention from negative material and biases in memory, although there may be some differences between the two clinical syndromes with regard to these cognitive processes (Craske et al., 2009; Gotlib & Joormann, 2010).

Cognitive biases as seen in depression and anxiety are likely to not only affect the processing of current and past experiences, but also the processing of prospective-oriented material, i.e., cognitions relating to the future. There is accumulating evidence that the same neural pathways are activated when imagining the future as when remembering the past (Byrne, Becker, & Burgess, 2007; Schacter, Addis, & Buckner, 2007). According to the concept of the “prospective brain” (Schacter et al., 2007), our ability to imagine and predict potential future events is based on stored information in our memory. In line with this model and given the fact that depression is associated with biases in the processing of both positive and negative memories (Gotlib & Joormann, 2010), one would expect depression to be associated with a bias in the processing of both positive and negative prospective-oriented material. Research on possible memory biases in anxiety has yielded mixed findings (Craske et al., 2009), thus prohibiting clear predictions with regard to prospective-oriented information processing in this disorder.

With regard to prospective positive and negative cognitions in depression and anxiety, MacLeod, Tata, Kentish, and Jacobsen (1997) have hypothesized that prospective cognitions may follow the same pattern as positive and negative affect as postulated by the tripartite model (Clark & Watson, 1991). The tripartite model proposes that negative affect is shared by both depression and anxiety, whereas absence of positive affect is specific to depression. In this model, positive affect is seen as a dimension of pleasurable engagement, level of energy and concentration, whereas negative affect is thought of as a dimension of unpleasurable engagement and subjective distress. These dimensions are theorized to include broad affective, cognitive, and motivational characteristics (Clark & Watson, 1991). MacLeod and Byrne (1996) and MacLeod et al. (1997) argue that affect is directly related to cognition and that positive and negative future-related cognitions may best be perceived as two separate dimensions of experience, differentially associated with depression and anxiety. Consequently, as depression is associated with increased negative affect and reduced positive affect it is also expected to be related to increased negative expectancies and decreased positive expectancies. In contrast, anxiety should only be associated with increased negative expectancies through the high negative affect component.

Research into prospective mental imagery has taken two main approaches. The first is the examination of the *deliberate* (as opposed to involuntary) generation of specific prospective images in response to set cues such as short sentences in the laboratory. Macleod, Rose, and Williams (1993) used an adaptation of the Autobiographical Memory Task (typically used to study overgeneral past memory in depression) where participants are required to generate as many positive and negative future events as possible. In this adapted fluency measure of future thinking, participants are presented specific time periods in the future and asked to generate experiences they are looking forward to and not looking forward to, for example, next year or in five years time. Time periods are presented verbally, one at a time, and participants are given a time limit of one minute to generate and say aloud as many responses as they can. The items generated by participants are written down by the researcher. An example of a deliberately generated positive future event reported by participants might be “taking a vacation,” whereas an example of a negative future event might be “getting a disease.” Although deliberately generated in the laboratory, these same events may also be experienced as involuntary future images. Number of responses generated per condition (i.e., future positive experiences *vs.* future negative experiences) counts as the outcome measure. Macleod et al. (1993) have found this fluency measure of future thinking to be effective in eliciting personally relevant responses. In a study with suicidal patients and nondepressed controls, Williams et al. (1996) reported that deficits in being able to recall specific past memories were associated with deficits in generating specific future images. MacLeod and Byrne (1996) further found that both anxious and anxious-depressed participants showed greater anticipation of negative experiences that might happen to them in the future than the control group using this task. Furthermore, as expected, only anxious-depressed participants showed lower anticipation of positive future-directed experiences. In a study investigating clinical depression and anxiety, MacLeod et al. (1997) found that participants with depression generated less positive prospective experiences than control participants. However, contrary to their expectations and contrary to the tripartite model, participants with anxiety (i.e., not those with depression) generated a greater number of prospective negative experiences compared to controls.

An alternative to the assessment of fluency has been to assess the vividness of imagery for prospective events generated in response to a set list. Using the Prospective Imagery Task (based on MacLeod & Byrne, 1996), Stöber (2000) investigated the vividness of prospective positive and negative mental imagery in non-clinical anxiety and depression. Examples from this set list are “you will do well on your course” for a positive prospective event or “you will be a victim of a crime” for a negative prospective event. In this study by Stöber, only depression (and not anxiety) showed a unique relationship with impoverished vividness of positive prospective events. Furthermore, only anxiety (and not depressed mood) was correlated with enhanced imagery for negative prospective events. Using the same measurement of the vividness of prospective events in a study with non-clinical participants with high or low levels of dysphoria (depressed mood), Holmes, Geddes, Colom, and Goodwin (2008) reported that high levels of dysphoria were associated with lower vividness of positive (but not negative) prospective images. These findings are also contrary to predictions based on the tripartite model or the model of the prospective brain. Taken together, predictions based on the tripartide model (Clark & Watson, 1991) and the model of the “prospective brain” (Schacter et al., 2007) have not entirely been able to explain the association between prospective imagery and depression and anxiety. In accordance with these two models, MDD has indeed been associated with lower vividness of positive prospective events (Holmes, Lang, Moulds, & Steele, 2008; MacLeod & Byrne, 1996; MacLeod et al., 1997; Stöber, 2000). Further, several studies have reported that anxiety is associated with a higher vividness of negative prospective images (MacLeod & Byrne, 1996; MacLeod et al., 1997; Stöber, 2000). However, contrary to predictions based on the models, several studies have reported that MDD is not related to higher vividness of negative prospective images (Holmes, Lang, et al., 2008; MacLeod et al., 1997; Stöber, 2000).

The second approach has been to explore *intrusive* involuntary prospective imagery for real-world events, i.e., images of the future which come to mind unbidden rather than those generated in response to set cues in the laboratory as in the studies discussed above. The Impact of Future Events Scale (IFES; Deeprose & Holmes, 2010) was designed to measure the impact of “pre-experiencing” in the form of intrusive prospective, personally-relevant imagery, assessed through a series of self-report questions. A positive correlation between current depressive symptomatology and IFES Total Score has been observed in a non-clinical sample, with a mild-dysphoric group showing significantly higher Total IFES scores than a non-dysphoric group (Deeprose & Holmes, 2010). Total IFES score has also been associated with risk for bipolar disorder in a non-clinical sample (Deeprose, Malik, & Holmes, 2011). These results raise the possibility that intrusive prospective imagery may be of relevance in depression as well as anxiety.

A growing body of recent research has documented the association between imagery and mental disorders (Brewin, Gregory, Lipton, & Burgess, 2010; Hirsch & Holmes, 2007; Holmes & Hackmann, 2004; Holmes & Mathews, 2010). Mental imagery has been shown to evoke greater emotional responses than language-based representations (Holmes, Lang, & Shah, 2009; Holmes & Mathews, 2010; Holmes, Mathews, Mackintosh, & Dalgleish, 2008). Furthermore, research suggests that prospective imagery affects future behavior. Libby, Shaeffer, Eibach, and Slemmer (2007) as well as Vasquez and Buehler (2007) have demonstrated that people are more motivated to accomplish future behavior and also to actually conduct the behavior in question if they imagine its successful completion from a third-person perspective rather than a first-person perspective. Holmes, Crane, Fennell, and Williams (2007) have shown that simulation of future events using imagery may be particularly concerning from a clinical perspective if the action is negative, such as in the case of “suicidal flashforwards” imagery (Crane, Shah, Barnhofer, & Holmes, in press).

The aim of the current study was to explore the relationship between positive and negative prospective mental imagery in patients with MDD and patients with anxiety disorders in comparison to healthy participants using established paradigms from experimental psychopathology research. First, we assessed vividness for *deliberately* generated mental images in response to a set list of prospective positive and negative events using the Prospective Imagery Task (Holmes, Lang, et al., 2008; Stöber, 2000). Ratings were also obtained for arousal as well as the estimated likelihood that each event would occur in the future. Second, we investigated the impact of *intrusive*, prospective imagery of personally-relevant real-world events among patients with MDD and anxiety and in comparison to healthy participants using the IFES (Deeprose & Holmes, 2010).

In accordance with findings reported above (Holmes, Lang, et al., 2008; MacLeod & Byrne, 1996; MacLeod et al., 1997; Stöber, 2000), we predicted that for deliberately generated images, only participants with MDD (i.e., and not those with anxiety disorders) would report lower vividness of positive prospective images as compared to healthy participants. Furthermore, we predicted that only participants with anxiety disorders (i.e., and not those with MDD) would report higher vividness of negative prospective images than healthy participants (Holmes, Lang, et al., 2008; MacLeod & Byrne, 1996; MacLeod et al., 1997; Stöber, 2000). Finally, we hypothesized that both participants with MDD and those with anxiety disorders would report a higher impact of *intrusive*, prospective images of personal events than the control group (Deeprose & Holmes, 2010).

## 2 Methods

### 2.1 Participants

The samples consisted of 24 patients with MDD, 27 patients with anxiety disorders, and 32 healthy control participants. Patients with MDD or anxiety disorders were recruited from outpatient psychiatric clinics of the University of Frankfurt. Diagnoses were determined by qualified clinicians using the Structured Clinical Interview for DSM-IV axis for I disorders (First, Spitzer, Gibbon, & Williams, 1996) and Axis II disorders (Pfohl, Blum, & Zimmerman, 1997). The SCID was used in assessing the clinical groups only. Patients were included if they met criteria for MDD or at least one anxiety disorder. Exclusion criteria were acute suicidality, depressive disorder with psychotic symptoms, bipolar disorder, organic psychiatric disorders, substance-abuse disorders, schizophrenia, schizoaffective disorders, and borderline personality disorder. An additional exclusion criterion for the group with anxiety disorders was meeting criteria for MDD. On the other hand, anxiety disorders were an exclusion criterion for the MDD group.

In the MDD group, 70.8% of the participants were diagnosed with MDD as the only diagnosis. The rest of the sample had one comorbid diagnosis (three patients were diagnosed with a comorbid somatization disorder, two with a comorbid pain disorder, one with bulimia nervosa, and one with an avoidant personality disorder). Participants with MDD had a mean age of 42.0 (*SD* = 11.3) and 58.3% of them were female.

In the group of anxiety disorders, participants were diagnosed with the following primary diagnoses: panic disorder with (14.8%) or without (33.3%) agoraphobia, generalized anxiety disorder (22.2%), social phobia (7.4%), posttraumatic stress disorder (7.4%), agoraphobia (3.7%), obsessive-compulsive disorder (3.7%), and specific phobia (7.4%). The majority of participants (66.7%) had no comorbid disorder. Participants with a comorbid disorder were diagnosed with either another anxiety disorder (22.2% of the total sample) or another comorbid diagnosis (11.1% of the total sample: one with bulimia nervosa, one with hypochondriasis, and one with narcissistic personality disorder). The mean age of participants in the anxiety group was 35.1 (*SD* = 9.6) and 74.1% were female.

Individuals in the control group were recruited through advertisement and were matched to the patient groups with respect to age and gender. Potential control participants were included after having been screened with the Hospital Anxiety and Depression Scale (Zigmond & Snaith, 1983) and provided that they had no prior history of MDD or anxiety disorders. Within this sample, the average age was 38.4 (*SD* = 13.1) and the percentage of female participants was 62.5%.

This study was approved by the medical ethics committee of the University of Frankfurt. Written informed consent was provided by all participants.

### 2.2 Measures

The *Hospital Anxiety and Depression Scale* (HADS) (Zigmond & Snaith, 1983) was used to assess anxiety (7 items) and depression (7 items). Authors of the HADS have recommended a score of above 10 for probable clinical anxiety or depressive disturbance, respectively. The HADS has demonstrated good reliability and validity properties (Herrmann, 1997). In the current study, the internal consistency reliability of the HADS anxiety subscale (*α* = 0.85) and depression subscale (*α* = 0.88) were satisfactory.

The *Prospective Imagery Task* (PIT; based on MacLeod & Byrne, 1996; Stöber, 2000) was used to measure imagery for 10 positive and 10 negative prospective events. As in Holmes, Lang, et al. (2008), subjects were asked to rate the vividness of prospective positive events (e.g., “You will have lots of energy and enthusiasm”) or negative events (e.g., “Someone close to you will reject you”) on a 5-point scale (1 = no image at all; 5 = very vivid). However, in addition to the vividness, in the current study, the PIT was modified to also include arousal associated with prospective images and the estimated likelihood that prospective images will occur in the future. Levels of emotional arousal were assessed in line with the study's focus on emotional disorder in order to explore whether participants in the clinical groups would report higher levels of emotional arousal associated with the vividness of positive or negative prospective imagery compared to controls, with whom the measure has been predominately utilized. Participants were first instructed to read a particular future scenario (e.g., “Someone close will reject you”) and to imagine the scenario happening to them. Then they were asked to rate the vividness of the scenario in question (“How vividly can you imagine this scenario?”). Then, they were asked to rate the arousal associated with each the scenario in question (“How emotionally aroused do you feel while having this image”). Finally they were asked to calculate how likely it is that that particular scenario might occur in future (“How likely is it that this scenario might happen to you in the future”). Rates of arousal and estimated likelihood were also rated on a 5-point scale. In the current study, the internal consistency of the PIT positive subscale was *α* = 0.89 for the measurement of vividness, *α* = 0.87 for the measurement of arousal, and *α* = 0.89 for the measurement of likelihood. The internal consistency of the PIT negative subscale was *α* = 0.83 for the measurement of vividness, *α* = 0.87 for the measurement of arousal, and *α* = 0.84 for the measurement of likelihood.

The *Positive and Negative Affect Schedule* (PANAS) (Watson, Clark, & Tellegen, 1988) was administered to separately measure both positive and negative affect. In the current study, the short version of the PANAS consisting of five items for positive affect and five items for negative affect was used (Thompson, 2007). Items are scored on a 5-point Likert-type scale ranging from *not at all* (1) to *extremely* (5). Participants were asked to anchor their responses to feelings during the last two months. The internal consistency reliability in the current study was *α* = 0.81 for the subscale of positive affect and *α* = 0.84 for the subscale of negative affect.

The *Impact of Future Event Scale* (IFES) (Deeprose & Holmes, 2010) was used to assess the impact of intrusive prospective, personally-relevant imagery. To encourage participants to respond on IFES in relation to idiosyncratic future events, participants were first asked “Please identify three future events which you have been thinking about by imagining over the past seven days (e.g., positive or stressful life events). For each event, please indicate whether your imagining of it was positive or negative.” Thus, participants could respond with solely positive or solely negative events, or a combination of both. Participants then responded to 24-items with the instructions “Below is a list of comments made by people about imagining events in the future. Please read each item, indicating how frequently each comment was true for you during the past 7 days due to imagining the future”. Items included “Pictures about the future popped into my mind,” “I tried not to think about the future” and “I had waves of strong feelings about the future.” Each item was anchored on a 5-point scale.

In scoring the IFES, the primary outcome variable is IFES Total Score which is the summation of responses to the 24-items. The secondary outcome variable is the number of negative events per individual, which is summed to create “IFES Negative Events”, i.e., the total number of events rated by the participant as negative. The total number of events provided by each participant is three. IFES has good internal consistency (Cronbach's alpha = 0.87) and adequate test re-test reliability (*r* = 0.73) (Deeprose et al., 2011). The internal consistency of the IFES in the current study was *α* = 0.90.

## 3 Results

### 3.1 Baseline characteristics

The three groups were comparable in terms of age, *F*(1,80) = 2.47, *p* = 0.17, and gender, *X*^2^(2, 83) = 1.54, *p* = 0.46.

Mean scores of each group on the depression and anxiety subscale of the HADS are shown in Table 1. Participants in the group of anxiety disorders reported significantly higher anxiety scores than both participants in the MDD group and control participants. Likely wise, participants with MDD had significantly higher depression scores than both participants with anxiety disorders and control participants.

There were significant differences between the groups with respect to positive and negative state affect. Compared to the control group, the two clinical groups reported lower scores of positive affect and higher scores of negative affect. Among the clinical groups, participants with anxiety disorders reported significantly higher scores of negative affect.

### 3.2 Mental imagery measures

Table 1 presents results for prospective mental images generated on the PIT. With regard to positive prospective scenarios (PIT positive), both clinical groups reported a poorer ability to vividly imagine prospective positive events as compared to the control group.^1^ The clinical groups also rated the likelihood of occurrence of positive events in the future as less likely than the control group. There were no differences among the groups with respect to arousal associated with prospective positive imagery.

As to prospective negative images (PIT negative), there were no differences between individuals with MDD and control participants. However, participants with anxiety disorders showed a greater ability to vividly imagine prospective negative scenarios than participants with MDD and control participants. Additionally, participants with anxiety disorders rated the likelihood of occurrence of negative scenarios in the future as more likely than control participants. Finally, participants with anxiety disorders reported higher arousal associated with prospective negative scenarios than control participants.

Concerning intrusive prospective imagery, both clinical groups reported higher IFES Total Scores, reflecting greater impact of intrusive prospective imagery of personally-relevant events as compared to the control group (IFES Total Score; Table 1). Both clinical groups reported a higher proportion of IFES Negative Events compared to controls. Within the clinical groups, there were no differences with regard to IFES Total Score or IFES Negative Events (Table 1).

## 4 Discussion

To date little research has examined prospective mental imagery in anxiety and depression, despite its clinical relevance to cognitive behavioral formulations of these disorders. In line with our predictions, we showed first that depressed patients, compared to controls, had impoverished imagery vividness when asked to deliberately imagine positive future events. This finding is clinically compelling as it suggests even if depressed people are asked to try to imagine a positive future, it is seen less clearly.

Second, as predicted results showed that patients with anxiety disorders (and not those with depression) imagined negative prospective images more vividly than the healthy participants. This is consistent with clinical reports by anxious patients being assailed by vivid imagined future threats. Importantly, this suggests that if given a trigger, such as a negative warning in a newspaper, people with anxiety would be susceptible to seeing the worst outcome more clearly and intensely than non-anxious individuals.

Our final key finding concerned intrusive, involuntary personal imagery rather than deliberate imagery. As predicted, data revealed that both patients with anxiety and depression reported a higher impact of *intrusive*, involuntary prospective images of personal events than the control group. This suggests that patients experiencing both disorders are more prone to unwanted images of events in the future springing to mind unbidden.

To the best of our knowledge, this is the first study to explore the role of mental imagery in prospective cognition using a convergent battery of measures to examine both deliberately generated and intrusive real-world mental imagery in clinical samples of MDD and anxiety disorders. A further strength is the use of age and gender-matched controls. However, the study has also several limitations. The cross-sectional design of the study does not allow for conclusions of causal relations between depression and anxiety and prospective mental imagery. The anxiety disorder sample was composed of patients with different anxiety disorders. However, there may be differences between individuals with different anxiety disorders with respect to prospective imagery. Future studies are needed to examine this issue. Further, no inter-rater reliability regarding the use of the SCID was assessed though all interviewers were conducted by qualified clinicians. Finally, use of medication at the time of assessment was not assessed and thus it could not be measured whether medication might have influenced prospective cognitions. Future studies may wish to select a wider range of imagery measures, including laboratory based tasks rather than questionnaire measures and also measure whether psychopfarmaca influences prospective cognitions.

Nonetheless, these findings offer new insight into the potential role of mental imagery in prospective cognition in patients with MDD or anxiety disorders. As in prior research (Holmes, Lang, et al., 2008; MacLeod & Byrne, 1996; MacLeod et al., 1997; Stöber, 2000), patients with MDD reported lower scores for vividness of prospective positive scenarios than control participants. Furthermore, patients with MDD did not report higher vividness of negative prospective images than control participants, also a finding consistent with previous literature (Holmes, Lang, et al., 2008; MacLeod et al., 1997; Stöber, 2000) and in contradiction with predictions based on the tripartite model (Clark & Watson, 1991) or the current conceptualization of the prospective brain (Schacter et al., 2007). However, it has been reported that observer-perspective images are common in individuals with MDD (Kuyken & Howell, 2006; Moulds & Williams, 2007). Based on findings that observer-perspective is associated with reduced emotional arousal (Holmes & Mathews, 2010), it might be that in our study patients with MDD may have used observer-perspective more often than patients with anxiety disorders and thus have damped vividness of negative events. Yet, future research should test this hypothesis while looking at perspective in relation to prospective images among patients with MDD and anxiety disorders.

Our result that patients with anxiety disorders reported more vivid images of negative prospective events than healthy participants is also in line with prior studies (MacLeod & Byrne, 1996; MacLeod et al., 1997; Stöber, 2000). However, the outcome that patients with anxiety disorders also reported impoverished vividness of positive prospective events is seemingly contradictory to previous results. MacLeod and Byrne (1996), MacLeod et al. (1997) and Stöber (2000) found that anxious participants did not show lower levels of positive future experiences. However, as mentioned above, the fluency measure of prospective thinking as utilized by MacLeod and Byrne (1996) and the PIT used here are intended to measure different outcomes (fluency, i.e., number of prospective events *vs.* vividness of prospective scenarios). Thus, our findings can rather be seen as an assessment of another dimension of prospective cognitions (i.e., imagery rather than just verbal thought). Although we used the same measure of prospective imagery as Stöber (2000) we studied a clinical sample compared to the non-clinical analogue population used previously which may account for the disparity in results. Finally, the difference with regard to positive prospective images remained significant between patients with anxiety disorders and healthy participants even after controlling for current depression symptomatology.

Our exploratory examination of the arousal of generating imagery on the PIT did not reveal any differences between clinical groups and controls with regard to positive prospective images. Further, patients with anxiety disorders reported significantly higher levels of arousal associated with negative prospective cognitions than control participants. This indicates that impoverished vividness of positive prospective images is not related to reduced emotional arousal associated with these images. However, enhanced vividness of negative prospective images was related to enhanced emotional arousal. Patients with anxiety disorders who reported enhanced levels of vividness of negative images than controls also reported higher levels of emotional arousal associated with these images than controls. The estimated likelihood that prospective images will occur in the future was associated with levels of both positive and negative prospective images. Both patients with MDD and anxiety disorders rated the likelihood of occurrence of positive prospective events as less likely than the control group as they also rated impoverished vividness of these prospective events than controls. Similarly, patients with anxiety disorders rated negative prospective events as more likely than controls. These results are in line with previous related finding. Warren, Zgourides, and Jones (1989) reported that perceived likelihood of negative outcomes predicted avoidance behavior in patients with anxiety disorders. MacLeod et al. (2005) found that perceived likelihood of positive events was associated with levels of hopelessness. Vincent, Boddana, and MacLeod (2004) reported that parasuicide patients estimated personal goals as less likely to be attained.

Our findings underscore implications for cognitive theory and cognitive behavioral therapies (CBT) of depression and anxiety regarding mental imagery. First, if a deficit in positive imagery is a deficit, a clinical implication might be promoting stronger deliberate positive imagery of the future may be of benefit in depression and anxiety disorders (Hackmann, Bennett-Levy, & Holmes, 2011; Holmes, Lang, & Deeprose, 2009; Holmes, Mathews, Dalgleish, & Mackintosh, 2006). As this was not an intervention-based study, we did not assess the impact of promoting deliberate positive imagery per se, but our findings suggest that future research in this area would be fruitful (Blackwell & Holmes, 2010; Lang, Blackwell, Hamer, Davison, & Holmes, submitted for publication). Positive imagery can be boosted in a number of ways from imagery work developing an idiosyncratic and positive image (e.g., one image of a positive self-nurturer in compassionate mind work Gilbert & Irons, 2004; Lee, 2005) to encouraging systematic training to be able to better imagine numerous positive future events (Blackwell & Holmes, 2010; Lang et al., submitted for publication).

Our results indicate that deliberately generated prospective negative images may be more closely related to anxiety than to depression but that both MDD and anxiety may be associated with intrusive prospective imagery of real-world events. While intrusive negative imagery has long been described in anxiety (Hirsch & Holmes, 2007; Holmes & Hackmann, 2004), intrusive negative imagery has only more recently been described as a clinical feature in depression (Patel et al., 2007; Williams & Moulds, 2007). Targeting negative intrusive imagery in depression in the way it is targeted in anxiety may open new treatment options (Brewin et al., 2009; Kandris & Moulds, 2008). Techniques that could be used to target negative imagery include imagery rescripting (Holmes, Arntz, & Smucker, 2007) as well as simple exposure (Kandris & Moulds, 2008). Targeting negative imagery has already been pivotal in the development of (CBT) for post-traumatic stress disorder where individual hotspots and flashbacks are a focus (Ehlers & Clark, 2000) and social phobia which included behavioral experiments to challenge socially phobic imagery (Clark et al., 2006). This targeting of negative imagery may usefully be extended to mood disorders (Holmes, Geddes, Colom, & Goodwin, 2008). Further, boosting positive imagery of the future provides a novel experimentally driven target for treatment innovation in depression.

## Conflict of interest

None.

## Figures and Tables

**Table 1:** Mood measures (HADS and PANAS) and mental imagery measure (PIT and IFES) for each group separately, with post hoc-comparisons between pairs of groups.

	1. Anxiety (*n* = 28) *M* (*SD*)	2. MDD (*n* = 24) *M* (*SD*)	3. Control (*n* = 32) *M* (*SD*)	ANOVA *F*(2,80)	Pairwise post hoc-test *p*-value		
					1 *vs.* 3	2 *vs.* 3	1 *vs.* 2
HADS: anxiety	13.3 (4.3)	9.5 (4.4)	6.9 (3.5)	18.47	**<0.001**	**<0.05**	**<0.01**
HADS: depression	9.6 (3.7)	12.9 (3.5)	3.5 (2.2)	64.31	**<0.001**	**<0.001**	**<0.01**
PANAS: positive affect	13.5 (3.8)	14.2 (3.8)	17.9 (2.5)	14.91	**<0.001**	**<0.001**	0.47
PANAS: negative affect	15.5 (4.8)	13.3 (4.0)	8.7 (3.0)	23.56	**<0.001**	**<0.001**	**0.04**
PIT positive							
Vividness	3.1 (0.8)	3.1 (0.7)	3.8 (0.6)	8.79	**<0.01**	**<0.01**	0.99
Arousal	3.4 (0.7)	3.5 (0.8)	3.4 (0.7)	0.08	0.70	0.93	0.78
Likelihood	2.7 (0.7)	2.7 (0.7)	3.5 (0.7)	13.59	**<0.001**	**<0.001**	0.78
PIT negative							
Vividness	3.6 (0.7)	3.2 (0.6)	3.2 (0.8)	3.06	**0.03**	0.96	**0.04**
Arousal	3.9 (0.7)	3.5 (0.7)	3.2 (0.9)	4.18	**<0.01**	0.31	0.09
Likelihood	2.8 (0.8)	2.5 (0.5)	2.2 (0.6)	6.75	**<0.01**	0.15	0.06

IFES Total Score	1.8 (0.7)	1.8 (1.0)	1.0 (0.4)	11.29	**<0.001**	**<0.001**	0.97
IFES Negative Events	1.5 (1.0)	1.42 (0.8)	0.8 (0.7)	5.14	**<0.01**	**0.01**	0.79

*Note*: HADS = Hospital Anxiety and Depression Scale; PANAS = positive and negative affect schedule; PIT = Prospective Imagery Task; IFES = Impact of Future Event Scale; all *p*-values in bold < 0.05.

## Footnotes

1 An Ancova while using HADS depression subscale as a covariate still revealed a significant difference between participants with anxiety disorders and healthy participants.
